# Diversity of *Ktedonobacteria* with Actinomycetes-Like Morphology in Terrestrial Environments

**DOI:** 10.1264/jsme2.ME16144

**Published:** 2017-03-17

**Authors:** Shuhei Yabe, Yasuteru Sakai, Keietsu Abe, Akira Yokota

**Affiliations:** 1 Graduate School of Agricultural Sciences, Tohoku University1–1 Tsutsumidori-Amamiyamachi, Aoba-ku, Sendai 981–8555Japan; 2 Hazaka Plant Research Center, Kennan Eisei Kogyo Co., Ltd.44 Aza-Inariyama, Oaza-Ashitate, Murata-cho, Shibata-gun, Miyagi 989–1311Japan

**Keywords:** *Ktedonobacteria*, *Chloroflexi*, *Ktedonobacteria*-specific primer

## Abstract

Bacteria with an actinomycetes-like morphology have recently been discovered, and the class *Ktedonobacteria* was created for these bacteria in the phylum *Chloroflexi*. They may prove to be a valuable resource with the potential to produce unprecedented secondary metabolites. However, our understanding of their diversity, richness, habitat, and ecological significance is very limited. We herein developed a 16S rRNA gene-targeted, *Ktedonobacteria*-specific primer and analyzed ktedonobacterial amplicons. We investigated abundance, diversity, and community structure in forest and garden soils, sand, bark, geothermal sediment, and compost. Forest soils had the highest diversity among the samples tested (1181–2934 operational taxonomic units [OTUs]; Chao 1 estimate, 2503–5613; Shannon index, 4.21–6.42). A phylogenetic analysis of representative OTUs revealed at least eight groups within unclassified *Ktedonobacterales*, expanding the known diversity of this order. Ktedonobacterial communities markedly varied among our samples. The common mesic environments (soil, sand, and bark) were dominated by diverse phylotypes within the eight groups. In contrast, compost and geothermal sediment samples were dominated by known ktedonobacterial families (*Thermosporotrichaceae* and *Thermogemmatisporaceae*, respectively). The relative abundance of *Ktedonobacteria* in the communities, based on universal primers, was ≤0.8%, but was 12.9% in the geothermal sediment. These results suggest that unknown diverse *Ktedonobacteria* inhabit common environments including forests, gardens, and sand at low abundances, as well as extreme environments such as geothermal areas.

Many studies have examined the relationship between the complex morphological differentiation of actinomycetes and their capacity to produce secondary metabolites (26). The class *Ktedonobacteria*, the members of which have an actinomycetes-like morphology, was recently proposed (5) and placed in the phylum *Chloroflexi* (41), which is known to be a deep-branching lineage of the domain Bacteria. This class currently contains only six named species in two orders: in *Ktedonobacterales*, *Ktedonobacter racemifer* SOSP1-21^T^ (isolated from soil under black locust in Italy; [5, 6]), which is the first pure culture in the class, *Thermosporothrix hazakensis* SK20-1^T^ (isolated from ripe compost in Japan; [41]), and *T. narukonensis* F4^T^ (isolated from fallen leaves deposited on geothermal soil; [43]); and in *Thermogemmatisporales*, *Thermogemmatispora onikobensis* ONI-1^T^ and *T. foliorum* ONI-5^T^ (isolated from fallen leaves deposited on geothermal soil; [42]), and *T. carboxidivorans* PM5^T^ (isolated from a geothermally heated biofilm; [19]). Other cultivated isolates for which valid names have not yet been proposed include ten isolates (strain SOSP series, from the same source as *K. racemifer* SOSP1-21^T^; [5]) in *Ktedonobacterales*; and four isolates (strains P-352, P-359, T104, and T81 from geothermal soils; [32]) and two isolates (strains BPP55 and PM6 from the same source as *T. carboxidivorans* PM5^T^; [19]) in *Thermogemmatisporales*. Furthermore, we identified five isolates (strains FA, FB, F7, MYC, and I1 from the same source as *T. narukonensis* F4^T^; [43]) within the genus *Thermosporothrix*.

The known *Ktedonobacteria* have a complex morphology and differentiation. All are Gram-positive and aerobic, and form branched mycelia with spores, similar to those of mycelia-forming actinomycetes. Moreover, all type strains sporulate via the formation of multiple exospores per cell by budding, which is unique among bacteria (40). Strains SOSP1-9, from the strain SOSP series, may form sporangia (5). Genome sizes are 13.7 Mbp in *K. racemifer* SOSP1-21^T^ (6), 7.3 Mbp in *T. hazakensis* SK20-1^T^ (27), and 5.6 Mbp in *T. carboxidivorans* PM5^T^ (Integrated Microbial Genomes & Microbiomes database: http://img.jgi.doe.gov/). The genome of *K. racemifer* SOSP1-21^T^ encodes 11,453 putative proteins, which is the largest number reported for a prokaryotic genome to date (6). The genomes of *K. racemifer* SOSP1-21^T^ and *T. hazakensis* SK20-1^T^ also contain 15 and 21 secondary metabolite-related gene clusters, respectively, as assessed by the antiSMASH online tool (https://antismash.secondarymetabolites.org/) (unpublished).

These unique morphological and genomic characteristics led us to speculate that this class may constitute a valuable new microbial resource for the discovery of novel compounds. We previously identified new secondary metabolites from *T. hazakensis* SK20-1^T^ (27, 28). However, in order to use a taxon as a source for screening novel compounds, it is important to know the diversity and habitat of the organisms in question.

Previous studies based on clone libraries or next-generation sequencing of the 16S rRNA gene of bacteria showed that environmental clone sequences assigned to *Ktedonobacteria* are prominent in extreme environments such as volcanic, Antarctic, and cave ecosystems (2, 12, 14, 17, 25, 29, 34, 38). A few studies have also investigated the detection of these clone sequences at low abundances in non-extreme environments such as sandy soils (1) and soils of the Flooding Pampa of Argentina (24). These studies did not focus on *Ktedonobacteria* in various habitats. Therefore, the abundance, diversity, richness, habitat preference, and community structure of the class are poorly understood.

In order to obtain detailed information on minor communities in samples from a range of environments, it is more effective to use taxon-specific primers than universal primers applicable to all bacteria when analyzing the pool of PCR amplicons. Specific primers may also be used for the rapid and sensitive screening of a particular taxonomic affiliation from among cultured organisms and microbial DNA. However, although many group-specific primers for 16S rRNA genes already exist, a *Ktedonobacteria*-specific (KS) primer has not yet been developed.

In order to obtain a tool for the rapid screening of sites at which *Ktedonobacteria* occur and to investigate their diversity and community structure *in situ*, we developed a KS primer for the 16S rRNA gene. We then surveyed the diversity and abundance of the bacteria in soils, sand, bark, geothermal sediment, and ripe compost using the Illumina MiSeq sequencing of amplicons with the specific primer.

## Materials and Methods

### Site description and sample collection

We collected eight samples from six sites: three forest soils, one garden soil, one sand, and one bark as common mesic terrestrial environments; and a geothermal sediment and compost as less common environments from which several ktedonobacterial strains have already been isolated.

#### Soil and bark samples

Forest soil samples 1A and 1B were collected from depths of 0–10 cm (1A, upper layer, 16°C) and 15–30 cm (1B, lower layer, 15°C) at a site under a cherry tree (*Cerasus jamasakura*) in the town of Murata, Miyagi Prefecture, northeastern Japan (38°07′36″N, 140°42′21″E) in May 2015. We removed the aboveground litter before sampling. Approximately 5 g of bark was collected from the cherry tree.

Forest soil sample 2 was collected from a depth of 0–10 cm (22°C) under large bamboo plants (Bambusoideae) in the town of Toyohashi, Aichi Prefecture, central Japan (34°47′49″N, 137°29′07″E) in May 2015.

The garden soil sample was collected from a depth of 0–10 cm (26°C) under a loquat tree (*Eriobotrya japonica*) in a campus garden at Tohoku University, Sendai City, Miyagi Prefecture (38°16′34″N, 140°52′22″E) in May 2015.

#### Sand sample

Sand was collected from a depth of 0–10 cm (24°C) on a riverbank in the town of Murata, Miyagi Prefecture (38°07′56″N, 140°42′55″E) in May 2015.

#### Geothermal sediment sample

Sediment was collected at the same site from which the *T. onikobensis* and *T. foliorum* isolates were previously collected (42) in the Jigokudani geothermal area, near Onikobe, in the Naruko hot springs region of Miyagi Prefecture (38°48′18″N, 140°40′24″E). We sampled fallen leaves deposited on steaming geothermal soil at a depth of 10–15 cm within the litter layer (72°C) in November 2015.

#### Compost sample

The sample was collected from ripe compost produced by a field-scale manure composter (Hazaka Plant, Kennan Eisei Kogyo, Murata Town, Japan; [39]) in May 2015. A species in the genus *Thermosporothrix* was previously isolated from compost produced by the same system (41).

All samples were collected in plastic press-seal bags with autoclaved scoops, transported to the laboratory, and stored at –80°C until DNA extraction and soil analyses. The characteristics of all samples are summarized in Table 1.

### Soil physicochemical analysis

Samples were dried at 105°C in an oven for 5 h in order to assess moisture content. The pH of a suspension of 10 g of wet soil in 100 mL of deionized water was evaluated with a pH electrode. Air-dried samples were sieved through a 2-mm mesh, and total C and N contents were measured with a CN analyzer (JM3000N/CN, J Science Lab, Kyoto, Japan). The results obtained are presented in Table 1.

### Design of a 16S rRNA gene-targeted KS primer

We designed a KS primer to amplify the 16S rRNA gene. We randomly selected 121 16S rRNA sequences (isolates and uncultured sequences, >1,200 bp and good quality) from each class in the phylum *Chloroflexi*, including *Ktedonobacteria*, from the Ribosomal Database Project II (RDP-II) (22). Multiple alignments of the sequences were performed using CLUSTALW (v. 1.83) (35), and consensus sequences were elucidated in BioEdit sequence alignment software (15). A KS forward primer was designed by comparing regions of nucleotide mismatches at the 3′ end of the oligonucleotide alignments of the consensus sequences by eye with those from other classes in the phylum (Fig. S1). We selected regions that had no mismatches at the 3′ end in the *Ktedonobacteria* class, but had mismatches in other classes. The primer was assessed *in silico* using the Probe Match tool in RDP-II (Differences Allowed: 0)

We named it KTED161F (*E. coli* position: 140–161). Since we were unable to find an appropriate KS reverse primer in the alignments, we used GNSB941R (*E. coli* position: 941–957), which was designed specifically for *Chloroflexi* (13).

We confirmed the specificity of the primer using all type strains within the class *Ktedonobacteria* and *Ktedonobacteria* isolates (strains FA, FB, F7, MYC, and I1). We performed the same tests using *Escherichia coli* NBRC3972, *Bacillus subtilis* NBRC3134, and *Micrococcus luteus* NBRC13867 as negative controls. In the results obtained, we confirmed that PCR bands were detected in the ktedonobacterial strains, but were absent in the negative controls.

### DNA extraction

DNA was isolated from 0.15 g of each sample using a Power Soil DNA Isolation Kit (Mo Bio Laboratories, Carlsbad, CA, USA) following the manufacturer’s protocols, and eluted in 50 μL of nuclease-free water. The quality of DNA was examined on agarose gels, and samples were quantified using the QuantiFluor dsDNA System (Promega, Fitchburg, WI, USA). DNA samples were stored at −20°C until further analyses.

### Amplification of the 16S rRNA gene by PCR for Illumina MiSeq sequencing

KS amplicons were generated by nested PCR and additional PCR was used for the barcoding of amplicons. The first round used the primers KTED161F and GNSB941R (Table 2). The predicted products (*ca.* 800 bp) were too long for sequencing on an Illumina MiSeq desktop sequencer and the detection sensitivity of one round of PCR was low; therefore, we conducted a second round (nested PCR), which also increased the likelihood of detecting products missed in the first round. The second round used KTED161F and UNIV538R; the latter targeted the 16S rRNA genes of the entire domain Bacteria (23) and was linked via an Illumina sequencing primer-binding site (Table 2). The third round used primers containing Illumina adapter sequences, an index sequence, and a sequencing primer-binding site without target gene-specific sequences. The index sequence was a unique sequence designed to differentiate sequencing reads from different samples (Table S1).

PCR was performed in 50 μL containing 1 μL of DNA (adjusted to 1 ng μL^−1^ for the first round, 1 μL of the first PCR product for the second round, and 1 μL of the second PCR product for the third round), 5 μL of 10×PCR buffer, 4 μL of dNTP mixture (2.5 mM each dNTP), 1 μL of each primer (0.2 μM), 38.5 μL of deionized distilled water, and 0.5 μL of Ex-Taq DNA polymerase (5 U μL^−1^) (Takara, Otsu, Japan). The first- and second-round PCR programs included an initial denaturation step at 94°C for 2 min; 30 cycles of denaturation at 94°C for 30 s, annealing at 55°C for 30 s, and extension at 72°C for 1 min; and a final extension at 72°C for 2 min. The third-round PCR program included an initial 94°C for 2 min; 10 cycles at 94°C for 30 s, 59°C for 30 s, and 72°C for 30 s; and a final 72°C for 5 min.

In order to evaluate the sensitivity of the new primer, we performed a control experiment using two rounds of PCR to amplify the 16S rRNA gene sequence (V1–V3) of the domain Bacteria. The first round used the universal primers UNIV27F and UNIV538R, linked via an Illumina sequencing primer-binding site (Table 2). The second round used primers containing Illumina adapter sequences, an index sequence, and a sequencing primer-binding site. The conditions for the first and second rounds were the same as those for the first and third rounds described above. All PCR products were separated by electrophoresis on 1.0% agarose gels, stained with a GelRed fluorescent DNA stain (Biotium, Hayward, CA, USA), and purified using a QIAquick Gel Extraction Kit (Qiagen, Valencia, CA, USA). The products were quantified with a QuantiFluor dsDNA System (Promega, Fitchburg, WI, USA), then mixed (50 ng each) and sent to FASMAC (Kanagawa, Japan) for Illumina MiSeq sequencing.

### Sequence analysis and evaluation of the new primer

We compared the KS amplicon sequences of the same samples between the new and universal primers. The raw reads obtained from the sequencer were sorted by the index sequence (one mismatch was allowed), trimmed for the primer sequences with the FASTX-Toolkit v. 0.0.13.2 tools (Gordon A., and G.J. Hannon. 2010. FASTX-toolkit. FASTQ/A short-reads preprocessing tools. [unpublished] http://hannonlab.cshl.edu/fastx_toolkit), filtered according to quality scores (threshold: 20) in Sickle v. 1.33 software (https://github.com/najoshi/sickle), and merged with forward and backward sequences by FLASH v. 1.2.10 software (21) at default conditions using the Quantitative Insights Into Microbial Ecology (QIIME) v. 1.8.0 software package (4). The universal amplicons were used as single reads. Processed high-quality reads were assigned to taxa by the RDP classifier (36) on the basis of RDP data, with a minimum confidence level of 80% using the RDP online tool (http://pyro.cme.msu.edu/index.jsp). Reads that were taxonomically unclassified at the domain level were removed. We evaluated the specificity of the new primer and the abundance of KS amplicons in the samples using the processed high-quality reads (Table 3).

### Phylogenetic and statistical analysis of KS amplicons

**Fig. 1:**
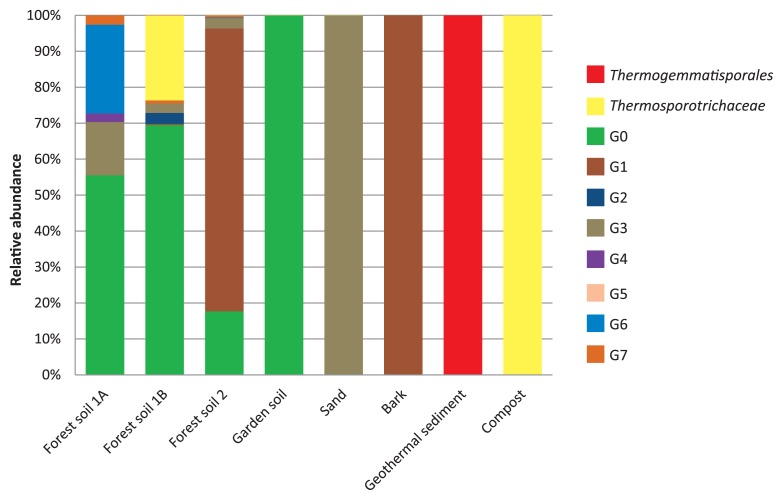
Comparison of ktedonobacterial community structures among soils, sand, bark, geothermal sediment, and compost. This figure shows OTUs that occupy >0.5% in at least one of the samples sequenced.

The high-quality reads that were assigned to *Ktedonobacteria* with high confidence (more than 70%) were used to analyze the diversity and phylogeny of the taxon. They were clustered into operational taxonomic unit (OTU) sequences that shared ≥97% similarity. We obtained representative sequences from each OTU using the functions pick_otus and pick_rep in QIIME and the UCLUST clustering algorithm (10) with default conditions. Ktedonobacterial singletons were not removed. The phylogenetic tree was constructed using OTUs that accounted for >0.5% of the relative abundance of *Ktedonobacteria* reads in each sample, together with the 16S rRNA genes of other *Ktedonobacteria* strains and clone sequences obtained from GenBank and RDP-II (8). Multiple alignments of the sequences were performed using CLUSTALW v. 1.83 (35), and the alignment was manually improved or unaligned regions trimmed with BioEdit software (15). The phylogenetic tree was constructed using the neighbor-joining method (30) in MEGA 5 software (33) with bootstrap values based on 100 replications (11). Evolutionary distances were computed using the Kimura two-parameter method (18). The taxonomic position of each OTU was assessed from the phylogenetic tree and compared with the ktedonobacterial composition of the samples. The compositions of the samples according to the clusters on the phylogenetic tree are compared in Fig. 1.

In the statistical analysis, we standardized the number of sequences per sample to 80,000 by random subsampling of the OTUs in order to avoid bias. Rarefaction curves, Shannon indices (31), and Chao 1 indices (7) were calculated using QIIME scripts (Table 4).

### Nucleotide sequence accession numbers

The sequencing data obtained in this study were deposited in the DDBJ Sequence Read Archive (DRA) under accession numbers DRR068692 to DRR068699 (universal amplicons) and DRR067880 to DRR067887 (ktedonobacterial amplicons).

## Results

### Physicochemical characteristics of samples

Differences among the eight samples were evident (Table 1). The temperature of the geothermal sediment sample (72°C) was markedly higher than those of the other samples (15–34°C). pH ranged from slightly alkaline in compost (pH 7.9) to slightly acidic in the other samples (pH 5.4–6.8). The total carbon contents of the upper-layer soils (forest soils 1A and 2) were high (7.56%–11.79%), whereas those of the lower-layer soils (forest soil 1B and garden soil) were low (0.32%–1.89%). The total nitrogen contents of the upper-layer soils (forest soils 1A and 2), the geothermal sediment, and compost were high (0.59%–2.4%), whereas those of the lower-layer soils (forest soil 1B) and sand were low (0.03%–0.08%).

### *In silico* analysis of KS primers

We selected a primer sequence (5′-ATACCGGBGMGAAAKYGYCGAC-3′) that showed dissimilarities to the 3′ end of the non-target sequences using the consensus sequence of the representative sequences in each class within the phylum *Chloroflexi* (Fig. S1). According to the Probe Match tool in RDP-II, this sequence completely matched 275 out of 1,459,456 sequences from the domain Bacteria, of which 273 (99%) were assigned to *Ktedonobacteria*. It matched 61% (273/448) of all known ktedonobacterial sequences in the database. However, since the important first two positions from the 3′ end of the primer matched 91% (403/448) of these sequences (Fig. S1), we decided to use this region as the KS primer, and named it KTED161F (Table 2).

The *Chloroflexi*-specific primer GNSB941R (5′-AAACCACACGCTCCGCT-3′) (25) completely matched 19,461 out of 1,459,456 domain Bacteria sequences, of which 19,244 (99%) were assigned to *Chloroflexi*. It also matched 94% (422/448) of all known ktedonobacterial sequences in the database.

We also evaluated the reverse primer UNIV538R (3′-GTATTACCGCGGCTGCTGG-5′) for second PCR (nested PCR), paired with KTED161F. UNIV538R completely matched 1,271,351 out of 1,459,456 domain Bacteria sequences and 89% (400/448) of all known ktedonobacterial sequences in the database.

### Evaluation of primer sets used to amplify 16S rRNA genes from environmental DNA samples

After the first KS amplification using the primer set KTED161F and GNSB941R, the PCR bands of the DNA samples obtained from the soils, sand, bark, and compost were either not visible or very weak, whereas those of the geothermal sediment sample were clearly visible. After second PCR using the primer set KTED161F and UNIV538R, the PCR bands of all samples were clearly visible. The PCR bands of all samples using the universal primer set (UNIV27F–538R) from the same DNAs were also clearly visible. After filtering, quality control, assembly of paired-end reads for ktedonobacterial amplicons, and removal of primers, chimeras, and low-confidence leads, 1,093,576 high-quality 16S rRNA sequence reads remained for ktedonobacterial PCR (81,296 to 250,365 per sample) in 1,430,264 raw reads and 749,093 for universal PCR (61,188 to 200,801 per sample) in 868,828 raw reads.

Most of the KS PCR amplicons (98.285%–99.996% of each total read) from each sample were assigned to *Ktedonobacteria* (Table 3). Very few (0.005%–0.80%) of the universal PCR amplicons were assigned to *Ktedonobacteria* (Table 3), except for 12.9% from the geothermal sediment. Thus, the primer set KTED161F-GNSB941R exhibited high specificity for the class *Ktedonobacteria*, which formed a small proportion of the samples tested in this study, except for the geothermal sediment. This low presence explains why the bands from first PCR were either not visible or very weak, despite the use of the KS primer, for all samples, except the geothermal sediment.

### Statistical and phylogenetic analysis of KS amplicons

We estimated the diversity of ktedonobacterial communities in samples from the KS amplicons. The number of OTUs markedly varied among the standardized sequences, ranging from 77 to 2,934 (Table 4). This analysis also indicated that the ktedonobacterial richness of these forest soils was higher than those of the other samples. The highest diversity was found in lower-layer forest soil (1B: Chao 1=5613, Shannon index=6.42, Simpson index=0.955), while the lowest was found in sand (Chao 1=506, Shannon index=0.03, Simpson index=0.003; Table 4).

**Fig. 2:**
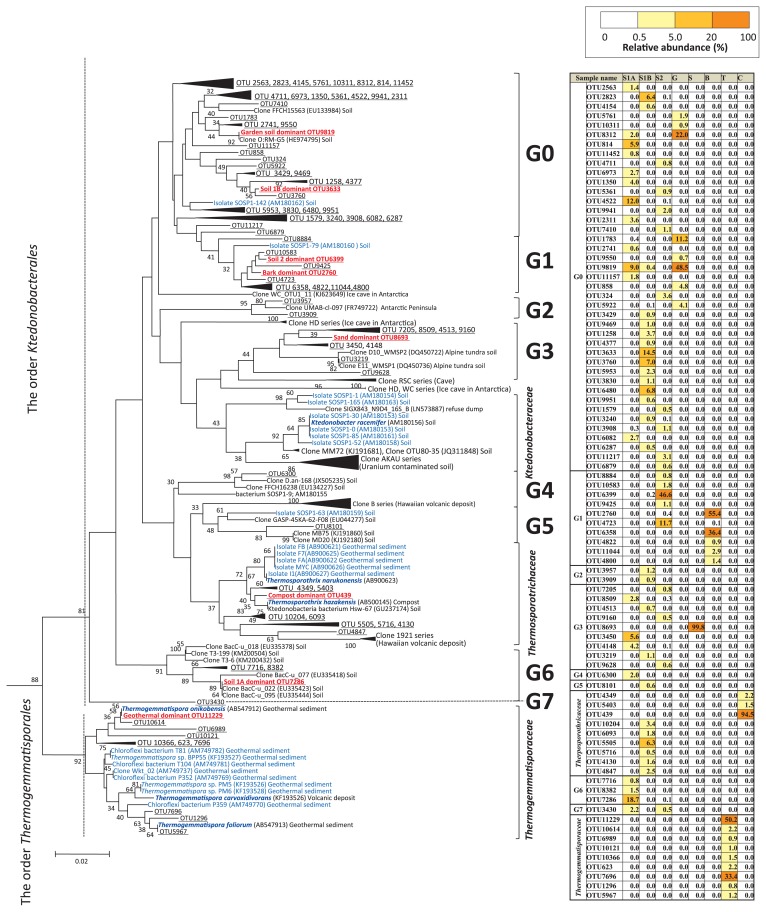
(A) Maximum likelihood phylogenetic analysis of representative 16S rRNA OTUs of ktedonobacterial amplicons from environmental DNA obtained from soils, sand, bark, geothermal sediment, and compost. The OTUs detected in this study are shown as underlines. The dominant OTU in each sample is shown in red. Cultured isolates of *Ktedonobacteria* are shown in blue (type strains are shown in bold blue). Numbers at nodes are bootstrap percentages based on 100 replicated data sets; only values >30% are shown. Scale bar, 2% sequence dissimilarity. *Caldilinea aerophila* (AB067647), *Litorilinea aerophila* (JQ733906), *Anaerolinea thermolimosa* (AB109437), *Anaerolinea thermophila* (AB046413), *Leptolinea tardivitalis* (AB109438), *Longilinea arvoryzae* (AB243673), *Bellilinea caldifistulae* (AB243672), *Levilinea saccharolytica* (AB109439), *Roseiflexus castenholzii* (CP000804), *Oscillochloris trichoides* (AF093427), *Chloroflexus aurantiacus* (D38365), *Chloroflexus aggregans* (CP001337), *Herpetosiphon geysericola* (AF039293), *Herpetosiphon aurantiacus* (CP000875), *Thermomicrobium roseum* (M34115), and *Sphaerobacter thermophilus* (AJ420142). *Aquifex pyrophilus* (M83548) and *Hydrogenobacter thermophilus* (Z30214) were used as an outgroup. (B) The relative abundance of each OTU within each community in the tree. The relative values of OTU abundances are indicated by fill intensities at the bottom right corner. Abbreviated sample names: S1A; Forest soil 1A, S1B; Forest soil 1B, S2; Forest soil 2, G; Garden soil, S; Sand, B; bark, T; Geothermal soil, C; Compost.

We constructed a phylogenetic tree (Fig. 2) by assigning the OTUs that accounted for >0.5% of the relative abundance in each sample to the three known families and eight unclassified *Ktedonobacterales* groups (G0–7) on the tree. Groups 1–7 were supported at >30% of bootstrap values and Group 0 at <30%. We used this phylogenetic tree to compare the ktedonobacterial community composition of the eight samples.

**Fig. 3:**
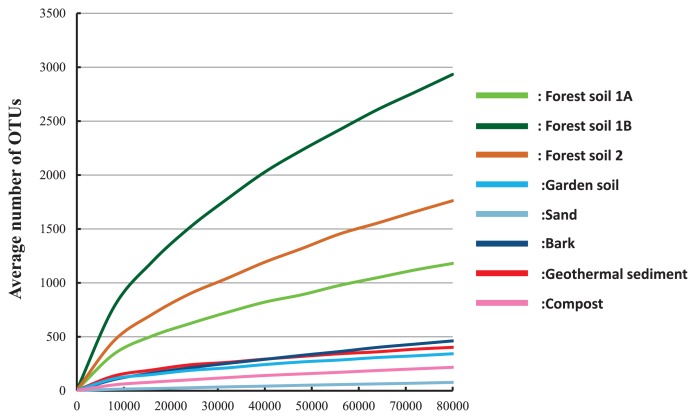
Rarefaction curves for reads normalized to 80,000 for each sample using 0.03 distance OTUs (*n*=10).

In the geothermal sediment sample, representative sequences from the family *Thermogemmatisporaceae* in the order *Thermogemmatisporales* dominated (93.5%; Fig. 2 and 3). Very few representative sequences from the other samples were assigned to this cluster.

In the compost sample, sequences from the family *Thermosporotrichaceae* in the order *Ktedonobacterales* were dominant (98.3%). In addition, OTU 439 was particularly dominant in this sample (94.5%), and, thus, diversity was low (Shannon=0.49; Table 4).

In the remaining samples (forest soils, garden soil, sand, and bark), most of the representative sequences were assigned to unknown *Ktedonobacterales* groups (52.4%–99.9%). The ktedonobacterial community in forest soil 1A was dominated by three unknown groups: group 0 (47.3%), group 6 (21.0%, including the dominant OTU 7286), and group 3 (12.6%; Fig. 1 and 2). In forest soil 1B, the community was dominated by group 0 (47.7%, including the dominant OTU 3633) and *Thermosporotrichaceae* (16.2%; Fig. 1 and 2). In forest soil 2, the community was dominated by group 1 (62.4%, including the dominant OTU 6399) and group 0 (14.0%). In garden soil, the community was dominated by group 0 (94.1%, including the dominant OTU 9819). The sand sample was dominated by group 3 (99.8%, including the dominant OTU 8693). In bark, the community was dominated by group 1 (97.1%, including the dominant OTU 2760; Fig. 1 and 2).

## Discussion

The class *Ktedonobacteria* was proposed ten years ago (5), and was more recently added to genetic databases. Therefore, few studies have discussed *Ktedonobacteria*. Prior to their proposal, environmental clones that have since been assigned to this class were described as unclassified bacteria or, in some cases, as unclassified *Chloroflexi*. For example, in 2006, ktedonobacterial clones that were detected in uranium-contaminated soil were reported as novel *Chloroflexi* clones with low homology to previously sequenced *Chloroflexi* (3), which were positioned within *Ktedonobacteraceae* in this study (Fig. 2). Likewise, environmental clones from an alpine tundra soil in the US Rocky Mountains (9) and from Hawaiian volcanic deposits (Clone 1921 series) (14) that were reported as new subdivisions of *Chloroflexi* were positioned within groups 3 and *Thermosporotrichaceae*, respectively, of *Ktedonobacteria* in this study (Fig. 2).

Since 2010, taxa within *Ktedonobacteria* are becoming more common in studies on microbial ecosystems in extreme environments. Many of these studies have described *Ktedonobacteria* as being among the most abundant types of bacteria in such environments. For example, they predominate in the cinder deposits of the Kilauea volcano (38), in lava caves (25), in high-elevation mineral soils (20), in soil from CO_2_ gas vents in the Calatrava volcanic field in Spain (29), in orthoquartzite caves in Venezuela (2), in the dark oligotrophic volcanic ice caves of Mt. Erebus in Antarctica (34), in soil from northern Victoria Land in Antarctica (17), and in geothermal soils on Pantelleria Island in Italy (12). Furthermore, most of the environments in which *Ktedonobacteria* have been found to predominate are acidic (2, 12, 17, 20, 29, 34); a survey of microbial ecosystems in the soil of Antarctica showed that they markedly decreased with increases in pH in the range of pH 5 to 7 (17).

The primary analytical method recently used to investigate the microbiota of various environments has changed from the 16S rRNA gene clone library method to the sequencing of millions of fragments using next-generation sequencing technologies from Roche and Illumina. These new methods allow researchers to investigate low-abundance taxonomic groups, and have facilitated the detection of ktedonobacterial phylotypes as a minor taxonomic group in terrestrial environments such as mesic soils (1, 24, 37, 44). These studies have led us to suspect that *Ktedonobacteria* only predominate in extreme acidic oligotrophic environments, such as volcanic fields and caves, and that they only occur at low abundances in more common terrestrial environments. The results of the present study have partially confirmed this: we found *Ktedonobacteria* in some common, mesic terrestrial environments at a low relative abundance (0.005–0.80%; Table 3), whereas they dominated in a hot geothermal sediment (12.9%; Table 3). However, the geothermal sediment was near-neutral (pH 6.8) and rich in organic matter (total C, 32.0%; total N, 1.6%); therefore, *Ktedonobacteria* may be dominant not only in acidic oligotrophic environments. In addition, this is the first time that *Ktedonobacteria* have been detected in bark.

This study is the first attempt to estimate their diversity and richness and to compare the community structure of their phylotypes in any environment using a specific primer. Our analysis showed that the diversity and richness indices of *Ktedonobacteria* in forest soils are very high (Table 4). In forest soils, the failure of the OTU rarefaction curves to plateau indicates that these communities were incompletely sampled (Fig. 3). Therefore, a large proportion of ktedonobacterial diversity remains to be sampled from these forest soils. Although these indices were lower in the other samples, each community was dominated by a different group (garden soil, G0; sand, G3; bark, G1; geothermal sediment, *Thermogemmatisporales*; compost, *Thermosporotrichaceae*; Fig. 1). These results suggest a diversity of phylotypes of *Ktedonobacteria* highly adapted to local environments, and so the overall diversity of the class may be extremely high. These results support our hypothesis that *Ktedonobacteria* are diverse in most environments.

The class *Ktedonobacteria* currently consists of two orders and three families. Almost all of the representative OTUs from soils, sand, and bark were not assigned to the known families. In our phylogenetic tree, OTUs were divided into eight groups, which lacked validly proposed cultured isolates (Fig. 1 and 2). Therefore, difficulties are associated with predicting their physiology, morphology, and ecological functions. However, strains SOSP1-142, 1-79, 1-9, and 1-63, isolated from soil in Italy (5), which have not yet been validly proposed as taxa, were positioned in groups 0, 1, 4, and 5, respectively, with low bootstrap values. Their known characteristics (5) are only that they were isolated at 30°C, form mycelia with spores, similar to typical actinomycetes, hydrolyze starch, casein, and gelatin, and are catalase-positive, Gram-positive, and resistant to some antibiotics. These characteristics may be common to the groups to which they belong. Groups 0 and 1 were the dominant groups in forest soils, garden soil, and bark (Fig. 1 and 2), implying that diverse undiscovered phylotypes with an actinomycetes-like morphology and not belonging to the class *Actinobacteria* may inhabit common terrestrial environments. All isolates previously assigned to *Ktedonobacteria* have an actinomycetes-like morphology (5, 19, 32, 40–43) even though they are distributed across two orders, three families, and groups 0, 1, 4, and 5 in the phylogenetic tree (Fig. 2). Therefore, it is possible that morphological features similar to those of actinomycetes are highly conserved across all phylotypes in *Ktedonobacteria*.

Groups 2, 3, and 5 include clones detected from extreme environments. Group 2 includes the unpublished clone UMAB-cl-097 (FR749722) from soil collected on the Antarctic Peninsula. This group formed a cluster with the OTUs from forest soil 1B at a high bootstrap value (95%), suggesting that the phylotypes of group 2 are not restricted to Antarctica.

Group 3 includes the clones D10_WMSP2 (DQ450722) and E11_WMSP1 (DQ450736), which were predominant in cold, acidic soil (pH 4.3–5.3, near 0°C) in the alpine tundra in the Rocky Mountains (9). These bacteria may play an active biogeochemical role in cold, anoxic soils (9). However, group 3 was dominated by OTUs from the sand sample (24°C), with markedly different characteristics from the cold soil collected in the alpine tundra. It also included 12.6% of the representative OTUs from forest soil 1A. Therefore, the phylotypes in this group occur in a wide range of environments, including extreme habitats and common environments such as forest soils and sand.

Group 5 includes the clone cluster (Clone B series) of the Hawaiian volcanic deposit (Fig. 2), predominantly comprising clones from unvegetated cinder deposits in the Hawaii Volcanoes National Park (38). Sequences most closely related to that of a gene for the large subunit of the carbon monoxide dehydrogenase (coxL) from *K. racemifer* dominated there (38), and some strains belonging to *Thermosporotrichaceae* and *Thermogemmatisporaceae* oxidize CO (19). However, it is not yet clear whether the phylotypes in group 5 have this ability because no strains belonging to group 5 have yet been isolated. In addition, 0.6% of the representative OTUs from forest soil 1B were affiliated with group 5. However, since this is a small proportion, and the other members of group 5 originated in a very different environment, it is difficult to make predictions about the characteristics of this group.

Groups 6 and 7 each formed a clade that was clearly distinguished from the other groups. Group 6 was predominant in forest soil 1A, at a relative abundance of 21.0%. It also included the clones BacC-u_018 (EU335378) and BacC-u_077 (EU335418), which had been found in soil with unsaturated C horizons in Tennessee, USA. Therefore, the phylotypes within group 6 may be widely distributed in soil ecosystems. Group 7 contained 2.2% of the representative OTUs in forest soil 1A.

In contrast, almost all the representative OTUs from the geothermal sediment and compost were assigned to known families in *Ktedonobacteria*. Compost was dominated by *Thermosporotrichaceae* (98.3%; Fig. 1). Species of *Thermosporothrix* form branched aerial mycelia with spores, and are Gram-positive, thermophilic, aerobic, and heterotrophic. *Thermosporotrichaceae* were isolated from compost produced by the same composting system (41); therefore, we expected this family to be predominant in the compost sample. However, *Ktedonobacteria* were rare in that sample (Table 3). *T. narukonensis* was isolated from fallen leaves deposited on geothermal soil and proposed as a novel species in the genus (43). These isolates commonly have an actinomycetes-like morphology and the ability to hydrolyze polysaccharides such as CM-cellulose, microcrystalline cellulose, and xylans (41, 43). Therefore, we consider this genus to contribute to the decomposition of polysaccharides in fallen leaves and wood fragments in compost and geothermal ecosystems. This family was also the predominant taxon in the OTUs of forest soil 1B (16.2%), indicating that it may include mesophilic species.

The geothermal sediment was the only sample in which *Ktedonobacteria* were predominant, at 12.9% relative abundance in the universal amplicons (Table 3). The KS amplicons of this sample were dominated by *Thermogemmatisporaceae* (93.5%) in *Thermogemmatisporales*. This order was detected almost solely in the geothermal sediment, although *Ktedonobacterales* are widely distributed in various environments. Furthermore, the sequences assigned to *Thermogemmatisporales* in the RDP database comprised only the three type strains and seven isolates described above and the environmental clone Wkt_02 (AM749737), all of which were isolated from the geothermal sediment. Therefore, the phylotypes in this order may be strongly adapted to environments characteristic of geothermal sediments. These isolates are commonly thermophilic bacteria with an actinomycetes-like morphology, which are able to grow at 40–74°C (19, 42). *T. onikobensis*, *T. foliorum*, and *T. carboxidivorans* hydrolyze cellulose and xylans (19, 42). *Thermobifida* and *Acidothermus* are well-known thermophilic cellulolytic actinomycetes, but cannot grow at >65°C. Therefore, phylotypes in the order *Thermogemmatisporales* may play a role in decomposing polysaccharides in very high temperature environments.

In summary, the results of the present study indicate that diverse phylotypes of the class *Ktedonobacteria* inhabit a range of environments, including mesic and extreme habitats, nutrient-rich and oligotrophic conditions, high and low pH, and high and low temperatures. This diversity suggests that these phylotypes produce unique metabolites, particularly secondary metabolites, in order to survive in this range of environments because secondary metabolism is commonly associated with complex differentiation similar to that of the actinomycetes (2). It also strongly supports our hypothesis that *Ktedonobacteria* may contain valuable new resources for the discovery of novel compounds.

Few strains in the class *Ktedonobacteria* been isolated and cultured to date. It is easy to cultivate strains in the genera *Thermosporothrix* and *Thermogemmatispora* because their growth is rapid: they form aerial mycelia in less than a week on common media such as ISP1 and ISP3 (41–43). In contrast, the growth of *K. racemifer* SOSP1-21^T^ is slow (5), but does not require special culture conditions. Therefore, few strains may have been isolated due to their very low relative abundance in common mesic environments.

This study provides an insight into ktedonobacterial abundance, diversity, richness, and habitats in several terrestrial environments. However, further studies are needed in order to fully reveal the diversity and abundance of this class. It is important to develop selective methods for culturing *Ktedonobacteria*, which will contribute to the investigation of this class as a valuable new microbial resource and also as a substitute for actinomycetes, and will reveal the roles they play in their ecosystems.

## Supplemental data



## Figures and Tables

**Table 1:** Sample details.

Sample name	Depth (cm)	Latitude, longitude	Temperature (°C)	Moisture content (%)	pH	Total carbon (%)	Total nitrogen (%)
Forest soil 1 (A)	0–15	38°07′36″N, 140°42′21″E	16	55.0	5.4	11.79	0.83
(B)	15–30		15	19.6	6.0	0.32	0.08
Forest soil 2	0–10	34°47′49″N, 137°29′07″E	22	23.3	5.8	7.56	0.59
Garden soil	10–20	38°16′34″N 140°52′22″E	22	9.3	5.9	1.89	0.31
Sand	0–10	38°07′56″N, 140°42′55″E	24	2.7	5.7	0.05	0.03
Bark	—	38°07′36″N, 140°42′21″E	—	21.3	5.4	48.92	0.84
Geothermal sediment	10–15	38°48′18.28N 140°40′24.23E	72	82.4	6.8	32.0	1.6
Compost	—	—	34	51.2	7.9	21.3	2.4

**Table 2:** Primer sequences used in this study.

Primer	Sequences (5′-3′)	Target taxon	References
**KTED161F:**	**ATACCGGBGMGAAAKYGYCGAC**	***Ktedonobacteria* (class)**	**This study**
GNSB941R:	AAACCACACGCTCCGCT	*Chloroflexi* (phylum)	(14)
UNIV27F	AGAGTTTGATCMTGGCTCAG	Bacteria (domain)	(16)
UNIV538R	GTATTACCGCGGCTGCTGG	Bacteria (domain)	(25)

MiSeq-adapter (5′–3′)1)			Index2)	Sequence site (5′-3′)3)	Target site
**Primer set for class** ***Ktedonobacteria*** **amplicons**					
1st PCR	F	—	—	—	KTED161F
	R	—	—	—	GNSB941R
2nd PCR	F	—	—	ACACTCTTTCCCTACACGACGC	KTED161F
	R	—	—	GTGACTGGAGTTCAGACGTGTG	UNIV538R
3rd PCR	F	AATGATACGGCGACCACCGCGCTCTACAC	Table S1	ACACTCTTTCCCTACACGACG	—
	R	CAAGCAGAAGACGGCATACGAGAT	Table S1	GTGACTGGAGTTCAGACGTGTG	—
**Primer set for domain Bacteria amplicons (universal)**					
1st PCR	F	—	—	ACACTCTTTCCCTACACGACGC	UNIV27F
	R	—	—	GTGACTGGAGTTCAGACGTGTG	UNIV538R
2nd PCR	F	AATGATACGGCGACCACCGCGCTCTACAC	Table S1	ACACTCTTTCCCTACACGACG	—
	R	CAAGCAGAAGACGGCATACGAGAT	Table S1	GTGACTGGAGTTCAGACGTGTG	—

1) Flow cell-binding site for Illumina MiSeq sequencing.

2) Unique sequence designed to differentiate sequencing reads from different samples. The sequence of each index is shown in Table S1.

3) Sequence primer-binding site for Illumina MiSeq sequencing.

**Table 3:** Specificities of primer sets and abundances of reads assigned to the class *Ktedonobacteria* by the RDP classifier.

	Forest soil 1 (A)	Forest soil 1 (B)	Forest soil 2	Garden soil	Sand	Bark	Geothermal sediment	Compost
*Ktedonobacteria* class-specific amplicons (primers KTED161F-GNSB941, KTED161F-UNIV538R)								
Domain Bacteria	127731	104688	250365	127779	90988	150662	81296	160067
Class *Ktedonobacteria*	125540	104333	250228	127465	90983	150611	81293	159997
% of reads in the class *Ktedonobacteria*	**98.285**	**99.661**	**99.945**	**99.754**	**99.995**	**99.966**	**99.996**	**99.956**
Universal amplicons (primers UNIV27F-UNIV538R)								
Domain Bacteria	77615	88875	79132	83650	61188	80793	77039	200801
Class *Ktedonobacteria*	4	741	15	12	47	57	9912	19
% of reads in the class *Ktedonobacteria*	**0.005**	**0.80**	**0.02**	**0.014**	**0.08**	**0.07**	**12.9**	**0.01**

**Table 4:** Ktedonobacterial diversity and richness values in samples.

	OTU richness			OTU diversity
		
	No. of OTUs	Chao 1	Simpson	Shannon
Forest soil 1 (A)	1181	2503	0.926	5.00
Forest soil (B)	2934	5613	0.955	6.42
Forest soil 2	1762	3947	0.754	4.21
Garden soil	343	742	0.698	2.52
Sand	77	506	0.003	0.03
Bark	462	1259	0.550	1.56
Geothermal sediment	402	746	0.634	2.30
Compost	217	806	0.109	0.49

The OTUs used in the statistical analysis were standardized to 80, 000 sequences.
