# Supplementary material for: Diversity of Ktedonobacteria with Actinomycetes-Like Morphology in Terrestrial Environments
Source: Microbes Environ. 2017 Mar 17;32(1):61–70. doi: 10.1264/jsme2.ME16144 (PMC5371077; doi:10.1264/jsme2.ME16144)
Supplement: Supplementary file 1 [file 32_61_s1.pdf]

## Supplemental data

Table S1 Index sequences for *Ktedonobacteria*-specific and universal PCR.

| Sample name | Forward (5′–3′) | Reverse (5′–3′) |
|---|---|---|
| Forest soil 1A | TCTCTCCG | AGGAGTCC |
| Forest soil 1B | CTCTCTAT | CATGCCTA |
| Forest soil 2 | CTAAGCCT | AGGAGTCC |
| Garden soil | GTAAGGAG | CATGCCTA |
| Sand | ACTGCATA | CATGCCTA |
| Bark | ACTGCATA | AGGAGTCC |
| Geothermal sediment | AAGGAGTA | CATGCCTA |
| Compost | CGTCTAAT | CATGCCTA |

Yabe et al

(A)

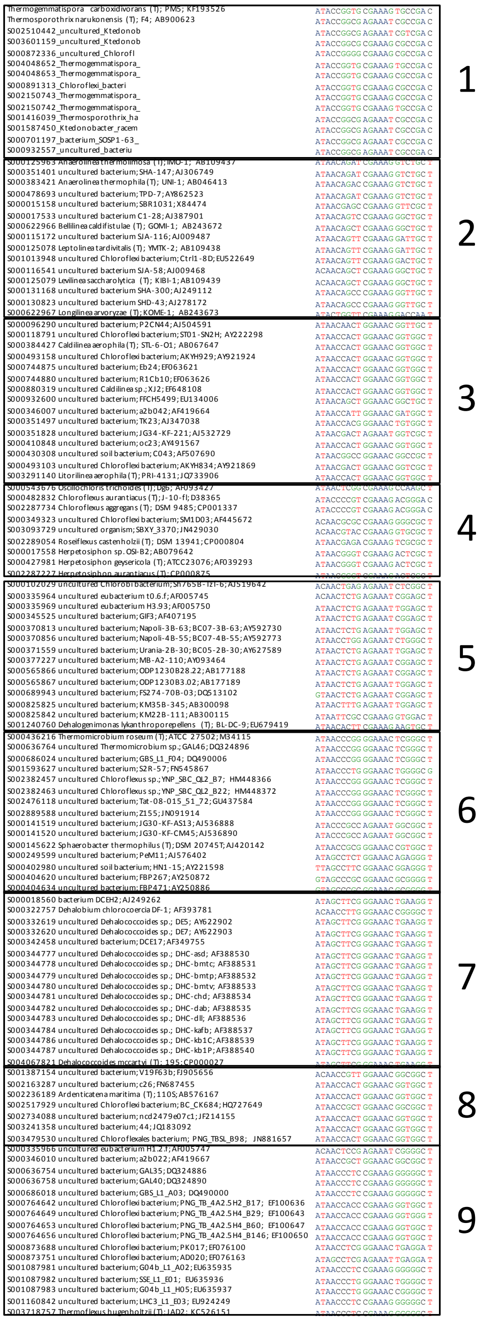


(B)

| | 5′- Consensus sequences -3′ |
|---|---|
| **1, *Ktedonobacteria*** | **ATACCGGBGMGAAAKYGYCGAC** |
| 2, *Anaerolineae* | AYAMYRRNYCGAAAGGDYHRM**T** |
| 3, *Caldilineae* | ATAACVRYBRGAAAYKRYBG**CT** |
| 4, *Chloroflexia* | AYAMCBNRNCGAAAGNBDVGMY |
| 5, *Dehalococcoidetes* | RYAAYHBDBMGAAAKNDSDR**CT** |
| 6, *Dehalococcoidia* | AYARCYTYGGGAAACYGRRG**ST** |
| 7, *Thermomicrobia* | DTAVCCBBBRGAAAYNSNGG**SK** |
| 8, *Ardenticatenia* | AYAACCRYTGGAAACGGYGG**CT** |
| 9, *Thermoflexia* | AYARCYNYBVGAAABBGNGGV**T** |
| *E.coli* ATCC11775 T (position 140-161) | ATAACTACTGGAAACGGTAG**CT** |

## Yabe et al

**Fig. S1 Alignments of class *Ktedonobacteria*-specific forward primer with other classes in the phylum *Chloroflexi*.** (A) Alignment of representative sequences in each class. (B) Alignment of consensus sequences in each class. Underlines indicate mismatched nucleotide to Ktedonobacterial consensus sequence.
